# Association of *MHTFR Ala222Val* (rs1801133) polymorphism and breast cancer susceptibility: An update meta-analysis based on 51 research studies

**DOI:** 10.1186/1746-1596-7-171

**Published:** 2012-12-07

**Authors:** Liwa Yu, Jianqiu Chen

**Affiliations:** 1 Department of General Surgery, the Secondary Hospital of Tianjin Medical University, Tianjin, 300211, China

**Keywords:** Polymorphism, Breast cancer, MTFHR, Ala222Val, Meta-analysis

## Abstract

**Background:**

The association between *MHTFR Ala222Val* polymorphism and breast cancer (BC) risk are inconclusive. To derive a more precise estimation of the relationship, a systematic review and meta-analysis was performed.

**Methods:**

A comprehensive search was conducted through researching MEDLINE, EMBASE, PubMed, Web of Science, Chinese Biomedical Literature database (CBM) and China National Knowledge Infrastructure (CNKI) databases before August 2012. Crude odds ratios (ORs) with 95% confidence intervals (CIs) were calculated to estimate the strength of the association.

**Results:**

A total of 51 studies including 20,907 cases and 23,905 controls were involved in this meta-analysis. Overall, significant associations were found between *MTHFR Ala222Val* polymorphism and BC risk when all studies pooled into the meta-analysis (Ala/Ala vs Val/Val: OR=0.870, 95%CI=0.789–0.958,P=0.005; Ala/Val vs Val/Val: OR=0.895, 95%CI=0.821–0.976, P=0.012; dominant model: OR=0.882, 95%CI=0.808–0.963, P=0.005; and recessive model: OR = 0.944, 95%CI=0.898–0.993, P=0.026; Ala allele vs Val allele: OR = 0.935, 95%CI=0.887–0.986, P=0.013). In the subgroup analysis by ethnicity, the same results were found in Asian populations, while no significant associations were found for all comparison models in other Ethnicity populations.

**Conclusion:**

In conclusion, our meta-analysis provides the evidence that *MTHFR Ala222Val* gene polymorphisms contributed to the breast cancer development.

**Virtual slides:**

The virtual slide(s) for this article can be found here: http://www.diagnosticpathology.diagnomx.eu/vs/1966146911851976

## Introduction

Breast cancer is the most common cancer and the main cause of cancer mortality in women. The etiology towards to this disease is poorly understood, some risk factors including familial history of the disease, age of menarche and of menopause, diet, reproductive history, high estrogen exposure as well as genetic factors may contribute to its development [1,2]. Studies suggest that the effect determined by low-penetrance genes, may provide a plausible explanation for BC susceptibility. Polymorphisms in genes are associated with a risk or protection against the disease. 5,10-methylenetetrahydrofolate reductase (*MTHFR*) is one important genes located at 1p36.3 [3]. *MTHFR Ala222Val* polymorphism has become the most commonly studied one, which has been considered to influence the enzyme activity of *MTHFR*[4]. The MTHFR 222Val/Val (homozygote) genotype results in 30% enzyme activity in vitro compared with the Ala/Ala wild-type [5]. Numerous epidemiological studies have evaluated the association between the *MTHFR Ala222Val* polymorphisms and BC risk. However, these studies have yielded conflicting results, partially because of the possible small effect of the polymorphism on BC risk and the relatively small sample size in each of published studies. The aim of this study is to derive a more precise estimation of these associations by performing this meta-analysis.

## Materials and methods

### Literature search

All studies that examined the association between the *MFTHR Ala222Val* polymorphism and BC were identified. A comprehensive search was conducted through researching MEDLINE, EMBASE, PubMed, Web of Science, China Biomedical Literature database (CBM) and China National Knowledge Infrastructure (CNKI) databases before August 2012. The search strategy included the combination of “breast cancer,” “breast neoplasm,” “methylene-tetrahydrofolate reductase,” “*MTHFR*,” “Ala222Val”, “rs1801133”, “variant,” and “polymorphism.” References of the retrieved articles were also screened. Non-familial case–control studies were eligible if they determined the distribution for this polymorphism in unrelated patients with breast cancer and in a concurrent control group of healthy subjects using molecular methods for genotyping. Of the studies with the same or overlapping data by the same investigators, we selected the most recent ones with the most subjects. We evaluated all associated publications to retrieve the most eligible literatures. The reference lists of reviews and retrieved articles were hand searched at the same time. We did not include abstracts or unpublished reports. When overlapping data of the same patient population were included in more than one publication, only the most recent or complete study was used in this meta-analysis.

### Inclusion and exclusion criteria

The following inclusion criteria were used to select literatures for the meta-analysis: (1) information on the evaluation of *MFTHR Ala222Val* polymorphism and BC susceptibility; (2)Only the cohort and case-control studies were considered;(3) sufficient genotype data were presented to calculate the OR with 95% CI. Major reasons for exclusion of studies were: (1) none-case–control studies; (2) reviews and duplication of the previous publication; (3) control population including malignant tumor patients; (4) no usable data reported.

### Data extraction

Two investigators reviewed and extracted information from all eligible publications independently, according to the inclusion and exclusion criteria listed above. An agreement was reached by discussion between the two reviewers whenever there was a conflict. The following items were collected from each study: first author’s surname, year of publication, ethnicity, total number of cases and controls with Ala/Ala, Ala/Val, and Val/Val genotypes, respectively. Different descents were categorized as Caucasians, Asians, and Mixed populations which included more than one ethnic descent. For case–control studies, data were extracted separately for each group whenever possible.

### Statistical analysis

The strength of the association between *MFHTR Ala222Val* polymorphism and BC risk was measured by ORs, whereas a sense of the precision of the estimate was given by 95% Cls. The significance of the summary OR was determined with a Z-test. We first examined *MFHTR Ala222Val* genotypes using co-dominant model (homogeneous co-dominant model: Ala/Ala vs Val/Val, heterogeneous co-dominant model: Ala/Val vs Val/Val), recessive (Ala/Ala vs Ala/Val + Val/Val), and dominant (Ala/Ala + Ala/Val vs Val/Val) genetic models. Then, the relationship between the allele and susceptibility to BC was examined (addictive model: Ala allele vs Val allele). Stratified analyses were also performed by ethnicities. A chi-square-based Q-statistic test and an *I*^*2*^-test test were both performed to evaluate the between-study heterogeneity of the studies.

Two models including the fixed-effects model and the random-effects model of meta-analysis were applied for dichotomous outcomes. The fixed-effects model assumes that studies are sampled from populations with the same effect size, making an adjustment to the study weights according to the in-study variance. The random-effects model assumes that studies are taken from populations with varying effect sizes, calculating the study weights both from in-study and between-study variances, considering the extent of variation, or heterogeneity. A P-value ≥0.10 for the Q-test indicated lack of heterogeneity among the studies, and so the summary OR estimate of each study was calculated by the fixed-effects modelm [6]. Otherwise, the random-effects model (DerSimonian and Laird method) was used [7]. *I*^*2*^ statistic can be used to quantify heterogeneity irrespective of the number of studies. The significance of the pooled OR was determined by the Z-test and P<0.05 was considered as statistically significant. Subgroup analyses were performed by ethnicity to explore the reasons of heterogeneity. Sensitivity analyses were performed to assess the stability of the results. To investigate whether publication bias might affect the validity of the estimates, funnel plot were constructed. An asymmetric plot suggests a possible publication bias. Funnel plot asymmetry was assessed by the method of Egger’s linear regression test, a linear regression approach to measure funnel plot asymmetry on the natural logarithm scale of OR. The significance of the intercept was determined by the t-test suggested by Egger (P<0.05 was considered representative of statistically significant publication bias). All statistical tests were performed with Stata (Version 12.0, Stata Corporation, College Station, TX), using two-sided P-values.

## Results

### Eligible studies

51 eligible studies on *MTHFR Ala222Val* genotypes and colorectal cancer were identified through literature search and selection based on the inclusion and exclusion criteria [8-58]. The publishing year of the studies was from 2002 to 2012. There were 25 studies of Caucasian, 19 studies of Asians and 7 studies of Mixed populations. In total, 20,907 BC cases and 23,905 controls were included in the meta-analysis. The selected study characteristics were summarized in Table 1.

**Table 1:** **The main characteristics of these studies and the distribution of MTHFR *Ala222Val *genotypes and alleles among cases and controls**

**First author [Inference]**	**Year**	**Ethnicity**		**Cases**			**Controls**		***HWE***
			**CC**	**CT**	**TT**	**CC**	**CT**	**TT**	
Sharp [8]	2002	Caucasian	30	19	5	25	21	11	0.103
Campbell [9]	2002	Caucasian	140	162	33	118	92	23	0.420
Semenza [10]	2003	Caucasian	42	58	5	112	111	24	0.643
Langsenlehner [11]	2003	Caucasian	208	222	64	215	215	65	0.333
Ergul [12]	2003	Caucasian	60	41	17	94	87	12	0.164
Shrubsole [13]	2004	Asian	374	555	183	387	577	196	0.442
Fo¨rsti [14]	2004	Caucasian	134	81	8	181	104	13	0.689
Lee [15]	2004	Asian	58	96	32	50	80	17	0.076
Grieu [16]	2004	Caucasian	166	141	27	242	259	50	0.100
Lin [17]	2004	Asian	43	38	7	173	145	24	0.389
Qi [18]	2004	Asian	42	104	71	59	105	54	0.593
Chen [19]	2005	Mixed	398	476	189	440	509	155	0.689
Kalemi [20]	2005	Caucasian	19	16	7	23	20	8	0.313
Deligezer [21]	2005	Caucasian	98	68	23	128	83	12	0.759
Justenhoven [22]	2005	Caucasian	249	247	61	261	279	93	0.193
Chou [23]	2006	Asian	73	51	18	132	120	33	0.475
Kalyankumar [24]	2006	Caucasian	45	37	6	61	31	3	0.693
Xu [25]	2007	Mixed	398	476	189	440	509	155	0.689
Hekim [26]	2007	Caucasian	22	16	2	38	26	4	0.872
Macis [27]	2007	Caucasian	14	20	12	28	41	11	0.511
Yu [28]	2007	Asian	56	54	9	225	170	25	0.336
Reljic [29]	2007	Caucasian	40	44	9	27	34	4	0.114
Inoue [30]	2008	Asian	239	120	21	393	226	43	0.178
Kotsopoulos [31]	2008	Caucasian	383	421	140	252	341	87	0.087
Suzuki [32]	2008	Asian	150	220	84	338	425	146	0.522
Cheng [33]	2008	Asian	185	133	31	268	221	41	0.624
Langsenlehner [34]	2008	Caucasian	51	43	11	40	48	17	0.685
Ericson [35]	2009	Caucasian	255	235	50	531	452	91	0.707
Gao [36]	2009	Asian	202	305	117	235	301	88	0.592
Ma [37]	2009	Asian	124	183	81	115	188	84	0.663
Platek [38]	2009	Mixed	429	446	119	788	795	219	0.398
Henrı′quez-Herna′ndez [39]	2009	Caucasian	52	65	18	107	138	47	0.823
Cam [40]	2009	Caucasian	48	49	13	47	42	6	0.398
Maruti [41]	2009	Mixed	133	139	46	301	284	62	0.672
Ma [42]	2009	Mixed	225	188	45	222	187	49	0.309
Li [43]	2009	Asian	38	17	10	90	50	3	0.187
Yuan [44]	2009	Asian	16	35	29	32	35	13	0.516
Jin [45]	2009	Asian	18	20	3	49	41	10	0.742
Bentley [46]	2010	Caucasian	346	402	191	429	529	205	0.060
Alshatwi [47]	2010	Asian	34	50	16	36	49	15	0.800
Sangrajrang [48]	2010	Asian	410	144	9	366	110	11	0.427
Weiner [49]	2010	Caucasian	399	364	74	386	326	66	0.808
Prasad [50]	2011	Asian	124	5	1	116	8	1	0.062
Batschauer [51]	2011	Caucasian	27	34	7	42	34	9	0.593
Mohammad [52]	2011	Asian	168	53	1	198	37	0	0.190
Naushad [53]	2011	Asian	185	56	3	205	39	0	0.175
Cerne [54]	2011	Caucasian	222	238	62	108	124	37	0.882
Akram [55]	2012	Caucasian	65	25	20	55	45	10	0.855
Barbosa [56]	2012	Mixed	76	83	17	87	70	19	0.389
Lajin [57]	2012	Caucasian	44	52	23	65	48	13	0.359
Jakubowska [58]	2012	Mixed	2032	2166	580	1447	1481	422	0.156

*HWE* Hardy–Weinberg equilibrium.

### Meta-analysis results

**Figure 1:**
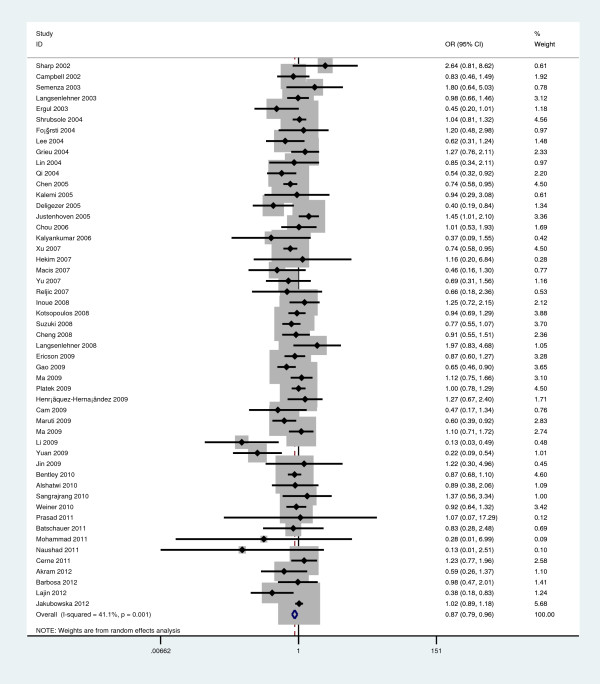
**Forest plot of overall breast cancer risk associated with the *MTHFR Ala222Val *polymorphism (Ala/Ala versus Val/Val).**

**Figure 2:**
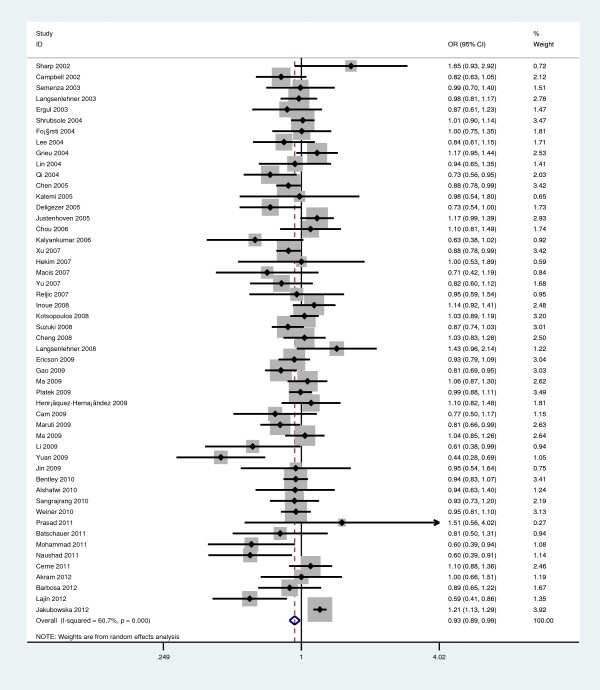
**Forest plot of overall breast cancer risk associated with the *MTHFR Ala222Val *polymorphism (Ala-allele versus Ala-allele).**

**Figure 3:**
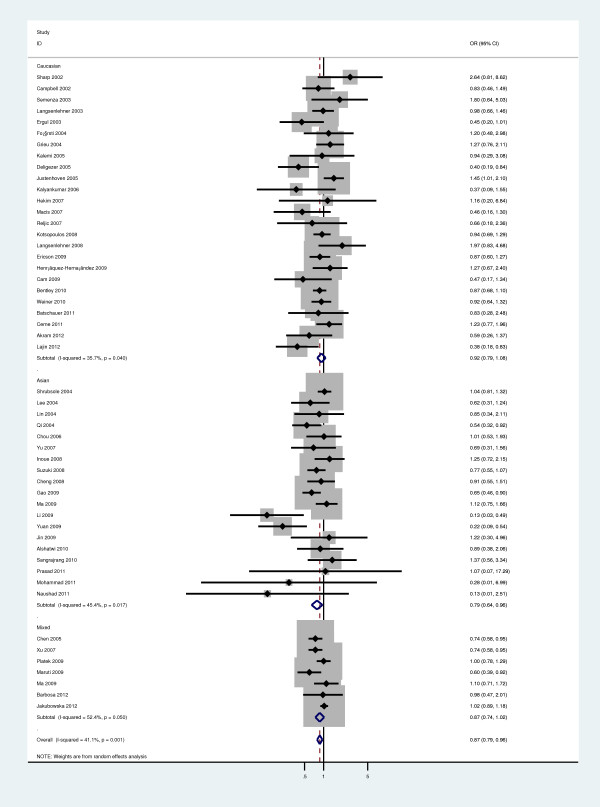
**Forest plot of a meta-analysis of the association between the *MTHFR Ala222Val *polymorphism and breast cancer susceptibility in Asians (Ala/Ala versus Val/Val).**

**Figure 4:**
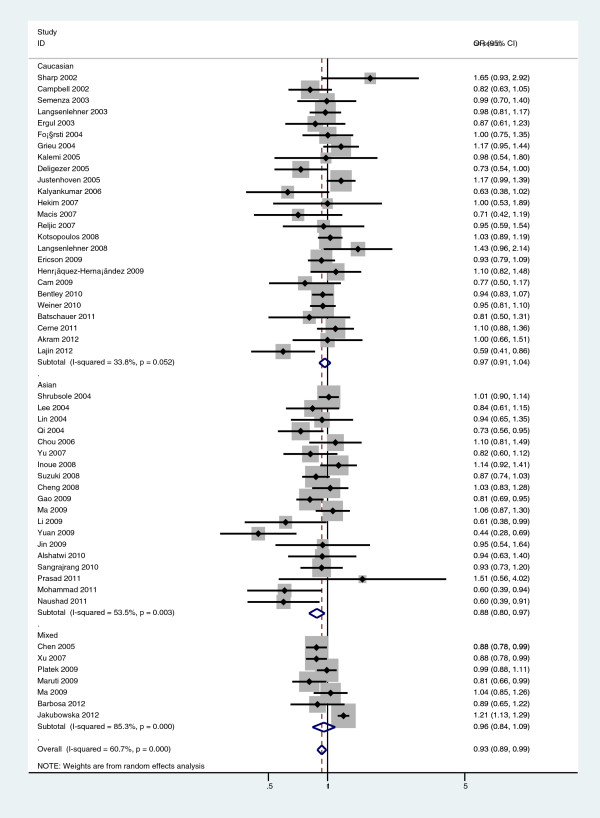
**Forest plot of a meta-analysis of the association between the *MTHFR Ala222Val *polymorphism and breast cancer susceptibility in Asians (Ala-allele versus Ala-allele).**

Overall, there was statistically significant difference in BC risk between the patients with Ala/Ala genotype and those with Val/Val genotype (OR=0.870, 95%CI=0.789-0.958, P=0.005; Figure 1). Similarly, significant associations were also found in the recessive model comparison (OR=0.944, 95%CI=0.898-0.993, P=0.026; Table 2) and dominant model comparison (OR=0.882, 95%CI=0.808-0.963, P=0.005; Table 2). Moreover, we found significant association between *Ala222Val* polymorphism and BC when examining the contrast of Ala versus Val (OR=0.935, 95%CI=0.887-0.986, P=0.013; Figure 2). In the stratified analysis by ethnicity, there was significant association between *Ala222Val* polymorphism and BC risk for Ala/Ala vs Val/Val comparison (OR=0.787, 95%CI=0.645-0.961, P=0.019; Figure 3), recessive model comparison (OR=0.890, 95%CI=0.799-0.991, P=0.034; Table 2), dominant model comparison (OR=0.826, 95%CI=0.703-0.972, P=0.021; Table 2) and Ala allele versus Val allele comparison (OR=0.877, 95%CI=0.801-0.960, P=0.008; Figure 4) among Asian populations. For Caucasian and Mixed populations, there was no significant association between *Ala222Val* polymorphism and breast cancer risk (Table 2).

**Table 2:** Main results of pooled odds ratios (ORs) with confidence interval (CI) in the meta-analysis

**Variables**	**No. of studies**		**Ala/Ala vs Val/Val**			**Ala/Ala vs Ala/Val**			**Ala/Val vs Val/Val**	
		**OR (95% CI)**	**P**_**h**_	**P**	**OR (95% CI)**	**P**_**h**_	**P**	**OR (95% CI)**	**P**_**h**_	**P**
Total	51	0.870(0.789 0.958)	0.001	**0.005**	0.969(0.923 1.016)	0.206	0.191	0.895(0.821 0.976)	0.021	**0.012**
Asian	19	0.787(0.645 0.961)	0.017	**0.019**	0.929(0.843 1.023)	0.212	0.132	0.865(0.753 0.993)	0.300	**0.039**
Caucasian	25	0.869(0.741 1.020)	0.040	0.319	1.004(0.921 1.095)	0.137	0.926	0.910(0.778 1.064)	0.031	0.238
Mixed	7	0.925(0.793 1.079)	0.050	0.087	0.958(0.898 1.022)	0.946	0.191	0.912(0.778 1.068)	0.050	0.253
**Variables**	**No. of studies**	**Ala/Val + Ala/Val vs Val/Va (dominant)**			**Ala/Ala vs Ala/Val + Val/Va (recessive)**			**Ala allele vs Val allele**		
		**OR (95% CI)**	**P**_**h**_	**P**	**OR (95% CI)**	**P**_**h**_	**P**	**OR (95% CI)**	**P**_**h**_	**P**
Total	51	0.882(0.808 0.963)	0.004	**0.005**	0.944(0.898 0.993)	0.055	**0.026**	0.935(0.887 0.986)	0.000	**0.013**
Asian	19	0.826(0.703 0.972)	0.075	**0.021**	0.890(0.799 0.991)	0.043	**0.034**	0.877(0.801 0.960)	0.003	**0.008**
Caucasian	25	0.916(0.790 1.063)	0.030	0.247	0.985(0.908 1.069)	0.141	0.720	0.883(0.805 0.968)	0.052	0.359
Mixed	7	0.888(0.758 1.041)	0.029	0.144	0.946(0.890 1.006)	0.773	**0.076**	0.957(0.838 1.094)	0.000	0.523

P_h_: P value of Q-test for heterogeneity test.

### Sensitivity analysis

In order to compare the difference and evaluate the sensitivity of the meta-analyses, we conducted one-way sensitivity analysis to evaluate the stability of the meta-analysis. The statistical significance of the results was not altered when any single study was omitted, confirming the stability of the results. Hence, results of the sensitivity analysis suggest that the data in this meta-analysis are relatively stable and credible.

### Publication bias

Begg’s funnel plot and Egger’s test were performed to assess the publication bias. The shape of funnel plots did not reveal any evidence of obvious asymmetry in all comparison models, and the Egger’s test was used to provide statistical evidence of funnel plot symmetry. The results of Begg’s test did not show any evidence of publication bias.

## Discussion

Breast cancer is currently the most frequently occurring cancer and the leading causes of cancer-related death among women in the world. Single nucleotide polymorphism (SNP) is the most common form of human genetic variation, and may contribute to individual’s susceptibility to cancer, however, the underlying molecular mechanism is unknown. Previous study suggested that some variants, especially those in the promoter regions of genes, may affect either the expression or activity levels of enzymes [59-61] and therefore may be mechanistically associated with cancer risk. Previous studies on the relationship between *MTHFR Ala222Val* polymorphisms and BC risk were contradictory. These inconsistent results are possibly because of a small effect of the polymorphism on BC risk or the relatively low statistical power of the published studies. Hence, the meta-analysis was needed to provide a quantitative approach for combining the results of various studies with the same topic, and for estimating and explaining their diversity.

Meta analysis has great power for elucidating genetic factors in cancer. On the bases of the character of cancer, the effect of one genetic component on the development of the disease can be easily masked by other genetic and environmental factors. A meta-analysis potentially investigates a large number of individuals and can estimate the effect of a genetic factor on the risk of the disease [62,63]. The present study included data from 51 association studies that had investigated the relationship between the *MTHFR Ala222Val* polymorphism and BC.

This present meta-analysis, including 20,907 cases and 23,905 controls, concerned the *Ala222Val* polymorphism of *MTHFR* gene and BC risk. In the meta-analysis, we found that the variant genotypes of the *MTHFR Ala222Val* polymorphisms were significantly associated with BC risk. Simultaneously, the same results presented in stratified analysis by ethnicity. We found that the variant genotype of the *MTHFR Ala222Val* polymorphism, in Asian populations, was associated with significant increase in BC risk. Although the *MTHFR Ala222Val* polymorphism may be associated with DNA repair activity, no significant association of the variant genotype with BC risk was found in Caucasian and Mixed populations, suggesting the influence of the genetic variant may be masked by the presence of other as-yet unidentified causal genes involved in colorectal cancer.

Some limitations of this meta-analysis should be acknowledged. First, our result was based on unadjusted estimates, while a more precise analysis should be conducted adjusted by other factors like diet habit, smoking, drinking status, environmental factors and so on. Second, in the subgroup analyses by ethnicity, relatively limited study numbers to perform ethnic subgroup analysis of mixed populations. Moreover, there are no American and African-American descent populations. Thus, additional studies are warranted to evaluate the effect of this functional polymorphism on BC risk in different ethnicities, especially in American, African-American and Mixed populations. In addition, our analysis did not consider the possibility of gene-gene or SNP-SNP interactions or the possibility of linkage disequilibrium between polymorphisms.

Despite of some limitations, this meta-analysis provided evidence of the association between the *MTHFR Ala222Val* polymorphisms and BC risk, supporting the hypothesis that *MTHFR Ala222Val* polymorphism contributes to overall BC risk. In subgroup analysis, the same results were found in Asian populations. In order to verify our findings, well-designed studies including different ethnic groups with a careful matching between cases and controls should be considered in future association studies to confirm the results from our meta-analysis. Moreover, further evaluating the effect of gene-gene and gene-environment interactions on the *Ala222Val* polymorphism and BC risk are necessary.

## Abbreviations

BC: Breast cancer; HWE: Hardy–Weinberg equilibrium; OR: Odds ratio; CI: Confidence interval; MTHFR: Methylenetetrahydrofolate reductase.

## Competing interest

Both authors declared that they have no conflict interest in relation to this study.

## Authors’ contributions

LY drafted the manuscript, and carried out the molecular genetic studies, participated in the sequence alignment and JC drafted the manuscript, carried out the molecular genetic studies, participated in the sequence alignment and reviewed the manuscript. All authors read and approved the final manuscript.
